# Biomass-Derived Amorphous Carbon with Intrinsic Nitrogen Doping for Hydrogen Peroxide Electrosynthesis

**DOI:** 10.1021/acsomega.5c13403

**Published:** 2026-04-27

**Authors:** Fellipe dos Santos Pereira, Victor Magno Paiva, Agnes Candido Teixeira, Marcelo Eduardo Huguenin Maia da Costa, Emanuel do Couto-Pessanha, Bojan A. Marinkovic, Natasha Midori Suguihiro, Pedro Nothaft Romano, Marco Aurélio Suller Garcia, João Monnerat Araújo Ribeiro de Almeida, Eliane D’Elia

**Affiliations:** 1 Department of Inorganic Chemistry, Universidade Federal do Rio de Janeiro (UFRJ), Avenida Athos da Silveira Ramos, 149, Cidade Universitária, Rio de Janeiro 21941-909, Brazil; 2 Nanotechnology Engineering Program, 28125 UFRJ, Avenida Horácio Macedo, 2030, Cidade Universitária, Rio de Janeiro 21941-972, Brazil; 3 Department of Physics 28099 Pontifícia Universidade Católica do Rio de Janeiro (PUC-RJ), Rua Marquês de São Vicente, 225, Gávea, Rio de Janeiro 22451-900, Brazil; 4 Department of Chemical and Materials Engineering, Pontifical Catholic University of Rio de Janeiro (PUC-Rio), Rio de Janeiro, RJ 22453-900, Brazil; 5 Department of Nanotecnology, UFRJ - Duque de Caxias, Professor Geraldo Cidade, Rodovia Washington Luiz, 19593, Duque de Caxias 25240-005, Brazil; 6 Programa de Pós-Graduação em Química (PGQu), 28125 UFRJ, Avenida Athos da Silveira Ramos, 149, Rio de Janeiro 21941-909, Brazil

## Abstract

Herein, amorphous carbon particles were synthesized from pumpkin seed biomass as a coproduct of a carbon quantum dot preparation route. The material was subsequently investigated as an electrocatalyst for the selective two-electron oxygen reduction reaction (2e^–^ ORR) toward hydrogen peroxide production. The results demonstrate selectivity and stability comparable to those of conventional commercial carbon, while being obtained through a simple waste-valorization strategy without additional activation or external doping steps. SEM analysis revealed fragmented particles with irregular morphology and predominantly external surface area, consistent with the low porosity quantified by N_2_ adsorption (14.2 m^2^ g^–1^; pore volume 0.00597 cm^3^ g^–1^), while XPS analyses demonstrated the presence of oxygenated surface functionalities and verified endogenous nitrogen incorporation (∼1%), indicating successful self-doping derived solely from the precursor composition. Rotating disk electrode experiments showed an onset potential of 0.738 V vs RHE, and Koutecký–Levich analyses indicated an electron transfer number close to 2, in contrast to Vulcan XC-72, which displayed mixed 2–3e^–^ behavior. Rotating ring-disc electrode measurements were also performed and confirmed high stability and selectivity, with an average H_2_O_2_ yield of ∼91.8% across 1.0–0.22 V and an average electron transfer number of 2.16. Additionally, chronoamperometry demonstrated outperforming compared to several reported carbon-based electrocatalysts. Thus, the results demonstrate that pumpkin seed-derived amorphous carbon is a sustainable and low-cost electrocatalyst with strong potential for decentralized H_2_O_2_ production via the 2e^–^ ORR pathway.

## Graphical abstract

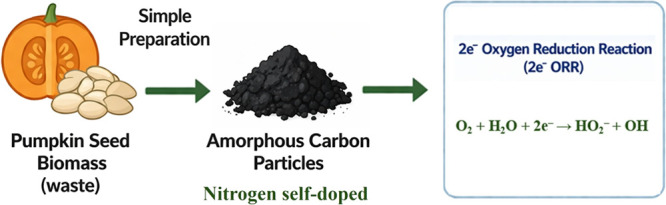

## 1 Introduction

Hydrogen peroxide (H_2_O_2_) is a highly versatile oxidizing agent whose physicochemical properties make it essential across a wide range of industrial and environmental applications. Beyond its classical use in pulp and textile bleaching, H_2_O_2_ is central to advanced oxidation processes, particularly Fenton and Fenton-like reactions, widely employed for the degradation of recalcitrant pollutants in wastewater treatment. Its relevance has further expanded into areas such as fuel cell technology, selective oxidation reactions in chemical synthesis, and antimicrobial processes aimed at bacteria and virus inactivation.^1–4^ In response to this broadening portfolio of applications, global demand is projected to increase steadily, reaching an estimated 5.7 million tons by 2027.^1^ However, the most widely used industrial process for peroxide production is the anthraquinone process, which, despite being well established and used, has disadvantages, such as huge energy consumption, environmental pollution, and safety problems.^2–4^ Alternatives have been used to produce H_2_O_2_ from H_2_ and O_2_. However, the use of H_2_ presents operational and safety challenges; its storage and transportation involve risk due to its high flammability and low ignition energy.^3^

In light of these challenges, and given the increasing demand for decentralized and on-site H_2_O_2_ production driven by safety, sustainability, and cost concerns, substantial research efforts have shifted toward electrocatalytic approaches based on the selective two-electron oxygen reduction reaction 2e^–^ ORR).^4–6^ Despite these advantages, important limitations persist. In alkaline media, the targeted pathway is inherently slow. Moreover, its selectivity can be compromised by competing reactions: oxygen may be reduced through the four-electron pathway and water oxidation can occur simultaneously, consuming charge and altering the local reaction environment, culminating in a reduced efficiency and selectivity of H_2_O_2_ production.^4,7^ Thus, the search for materials that exhibit selectivity for the 2e^–^ process and offer high conductivity has become the focus of several studies. The most widely used catalysts are platinum-based; nevertheless, the use of these materials is disadvantageous due to the high cost of the metal, low availability, toxicity, and low chemical resistance, including its high selectivity for the 4e^–^ pathway.^8,9^

In this context, the development of alternative catalysts that are low-cost, sustainable, and readily available is essential. Among these, carbon-based materials, particularly those derived from biomass, stand out, as they not only meet these requirements but also offer a large specific surface area, tunable morphology, high electrical conductivity, and low toxicity.^10,11^ When biomass is employed as the precursor, an additional advantage arises from its intrinsic heteroatom content. Owing to its naturally rich chemical composition, biomass typically contains substantial amounts of elements such as nitrogen, phosphorus, sulfur, and trace metals, enabling endogenous doping during carbonization.^11–13^ Additionally, the use of biomass also promotes the valorization of agricultural residues, which are generated worldwide in massive quantities, aligning with the principles of a circular and low-carbon economy by converting waste into high-value materials. Moreover, biomass-derived carbons can be produced at high yield and low cost, offering a clear advantage over conventional carbon synthesis routes – such as those used for graphene or carbon nanotubes – which are expensive, difficult to scale, and frequently dependent on toxic, environmentally harmful, and costly chemical precursors for heteroatom doping.^9,11–14^

Nanomaterials and biomass-derived carbon composites designed for the peroxide-selective oxygen reduction pathway have already been investigated in several studies. Both classes of materials have shown highly promising performance, in many cases achieving efficiencies that surpass those of commercial Pt/C catalysts. For example, Das et al.^15^ highlight the advantages of biomass-derived hierarchically porous carbon electrocatalysts for the 2e^–^ ORR, noting their intrinsic heteroatom doping (N, P, S, etc.) and outstanding physicochemical properties that support efficient H_2_O_2_ generation while avoiding noble-metal complexity. Similarly, Sun et al.^16^ produced a codoped N/O porous carbon catalysts obtained via biomass-based ZIF-67 precursors, which delivered ∼83% selectivity toward the 2e^–^ ORR and an H_2_O_2_ production rate of approximately 2,909.8 mmol g^–1^ h^–1^ at 0.36 V (vs RHE). These and other studies demonstrate that biomass-derived and nanostructured carbons represent a scalable, low-cost, and environmentally advantageous alternative to platinum-based catalysts for electrochemical H_2_O_2_ production, with strong potential for decentralized or on-site generation systems.

Based on the above discussion, in this work, pumpkin seeds (*Cucurbita maxima*), a widely available agro-industrial residue, were selected as the biomass precursor for carbon material synthesis. These seeds are typically discarded despite being produced in large quantities worldwide, particularly in food processing industries. Their rich biochemical composition, including proteins, lipids, and mineral elements such as nitrogen, phosphorus, and magnesium, makes them an excellent source for heteroatom-doped carbon materials.^17–20^ More importantly, however, is to highlight that the carbon material used in this study was obtained as a coproduct from a previous synthesis of carbon quantum dots (CQDs), which our group successfully applied as corrosion inhibitors.^21^ This dual valorization approach not only enhances the sustainability of the process by maximizing resource efficiency but also aligns with zero-waste principles. Building on these findings, the resulting porous carbon was further explored as an electrocatalyst for the selective 2e^–^ ORR toward hydrogen peroxide production with 91.8% of selectivity and a high H_2_O_2_ production rate of 612.7 mmol.g^–1^·h^–1^.

## 2 Materials and Methods

### 2.1 Materials and Chemicals

The pumpkin seeds used were obtained from a local market. Vulcan XC-72 (commercial electrocatalyst, E-TEK). Phosphoric acid 85% (Isofar), 0.22 μm filtration membrane (KASVI), 8 μm filter (KASVI), and 1 kDa dialysis membrane (PUR-A-LYZER) were used in the experiments.

### 2.2 Carbon-Based Material Synthesis

As mentioned previously, the carbon material used in this study was obtained as a coproduct during the synthesis of CQDs from pumpkin seeds.^21^ The procedure was carried out as follows: five grams of pumpkin seeds were ground using a blender and transferred to a round-bottom flask along with 5 mL of concentrated phosphoric acid. The flask was placed in an oil bath and heated at 200 °C for 8 h under reflux using a condenser. After cooling, 100 mL of deionized water was added to the reaction mixture. The resulting suspension underwent filtration, dialysis, and cold drying. To isolate the CQDs, the suspension was first filtered through 8 μm filter paper, followed by a 0.22 μm membrane, and then subjected to dialysis. The CQDs were subsequently collected and studied, as reported in a previous work where they were applied as corrosion inhibitors. In the present study, attention was directed to the carbon-rich fraction retained by the filters. Before dialysis, the crude suspension was centrifuged at 4000*g* to separate the amorphous carbon particles, which were precipitated. The collected solid was washed repeatedly (five times) with deionized water and ethanol using centrifugation to remove residual impurities. This amorphous carbon fraction was then dried and subsequently used as the electrocatalyst material in the present study.

### 2.3 Carbon-Based Material Characterization

For morphological analysis, the carbon particles were examined by scanning electron microscopy (SEM). Structural and surface properties were investigated using X-ray diffraction (XRD) and nitrogen adsorption–desorption isotherms (BET method). Thermogravimetric analysis (TGA) was employed to assess the thermal stability and composition of the materials. Additionally, the chemical composition and surface functionalities were analyzed by X-ray photoelectron spectroscopy (XPS).

Scanning electron microscopy images were acquired using a FEG-SEM JEOL JSM-IT700HR microscope, operated at 5 kV with secondary electron detection. X-ray photoelectron spectroscopy was conducted using an ultrahigh vacuum system (Specs System equipped with a Phoibos 150 Hemispherical analyzer) with a nonmonochromatic Al Kα X-ray source (hν = 1486.6 eV), operating at 15 kV and 20 mA. The survey scan covered an energy range from 0 to 1100 eV, while high-resolution spectra were obtained based on the sample composition at an energy setting of 30 eV.

### 2.4 Electrochemical Measurements

Electrochemical measurements were performed on an Autolab PGSTAT302N with FRA32 M module (Metrohm, The Netherlands) connected to a computer with NOVA 2.1.7 software. Rotation rates of the rotating ring-disc electrode (RRDE) were controlled by an Autolab Rotator (Metrohm, The Netherlands). A conventional three-electrode electrochemical cell with Ag/AgCl (3 M KCl) as the reference electrode, a Pt wire as the counter electrode, and an Autolab RRDE electrode with GC/Pt as the working electrode were used for the electrochemical measurements. For the modification, a suspension was prepared by mixing 2.5 mg of the electrocatalyst, 0.7 mL of deionized water, 0.5 mL of methanol, and 0.05 mL of Nafion 5.0 wt %. The suspension was left in an ultrasonic bath for 1 h and then for an additional 20 min before use. Electrochemical experiments were performed in a 0.1 mol·L^–1^ KOH solution. Cyclic voltammograms were performed under N_2_ and O_2_-saturated conditions, and polarization curves for ORR were performed in an O_2_-saturated solution. During the LSV test, the ring electrode voltage was fixed at 0.6 V vs Ag/AgCl to oxidize the H_2_O_2_ generated at the disk electrode. The electrocatalyst performance for ORR was measured by conducting LSV at 5 mV.s^–1^ in the potential range of 0 to −0.8 V vs Ag/AgCl.

The number of electrons was calculated by the Koutecky–Levich (K-L) equation, using the following equation:^22^

$$\frac{1}{j}=\frac{1}{j_{k}}+\frac{1}{B\omega^{1/2}}$$ 1

$$B=0.2nFD^{2/3}C_{0}\nu^{-1/6}$$ 2

where *j* and *j*_*k*_ are the current density and kinetic current density, respectively (mA cm^–2^), *n* is the number of electrons transferred in the overall reaction, *F* is Faraday’s constant (96485 C mol^–1^), *A* is the geometric area of the disk (0.196 cm^2^), *D* is the diffusion coefficient of O_2_ in the electrolyte (1.76 × 10^–5^ cm^2^ s^–1^) *C*_0_ is the solubility of O_2_ in the electrolyte (1.103 × 10^–6^ mol L^–1^), ν is the kinematic viscosity of the electrolyte, and ω is the angular frequency of rotation. It is important to note that, given the known limitations of the K-L method,^23^ RRDE measurements were also performed to provide additional insight into the ORR pathway.

The H_2_O_2_ selectivity, the Faradaic efficiency (FE), and the number of transferred electrons were calculated according to the measured disk current (I_D_) and ring current (I_R_).^24^ N is the current collection efficiency of the Pt ring, which was determined to be 0.25.

H2O2(%)=200%×IrNID+IRN 3

$$\mathrm{EF}=\frac{i_{R}}{|i_{D}|N}\times 100$$ 4

n=4IDID+IRN 5

Electrochemical impedance spectroscopy (EIS) was performed in a frequency range of 0.01 Hz–10 kHz with a perturbation amplitude of 10 mV at a rotation of 1600 rpm in 0.1 mol·L^–1^ KOH electrolyte saturated with O_2_. The electrochemical double layer capacitance (Cdl), commonly proportional to the electrochemical surface area (ECSA), was determined from the EIS data recorded in the nonphased region by eqs 6 and 7, 7

$$ECSA=A_{geo}\times \frac{Cdl}{Cs}$$ 6

$$Cdl=\frac{1}{2\pi fmaxRct}$$ 7

## 3 Results and Discussion

As can be seen in Figure 1, the structure of the carbon particles was investigated by SEM Figure 1A shows that the resulting material presents fragmented particles with irregular morphology. Interestingly, however, in the higher-magnification image (Figure 1B), rough, wavy, and partially smooth surfaces with low porosity can be identified, demonstrating that the resulting material exhibits characteristics of dense, slightly porous carbon. It is important to emphasize that the carbon material investigated in this work is not produced through a dedicated catalyst synthesis, but rather arises as a coproduct during the preparation of carbon quantum dots from pumpkin seed biomass. This strategy represents an integrated biomass valorization approach, in which multiple functional carbon materials are obtained from a single synthetic route. Instead of being discarded as residual solid waste, this fraction was isolated and directly employed as an electrocatalyst for the ORR.

**Figure 1:**
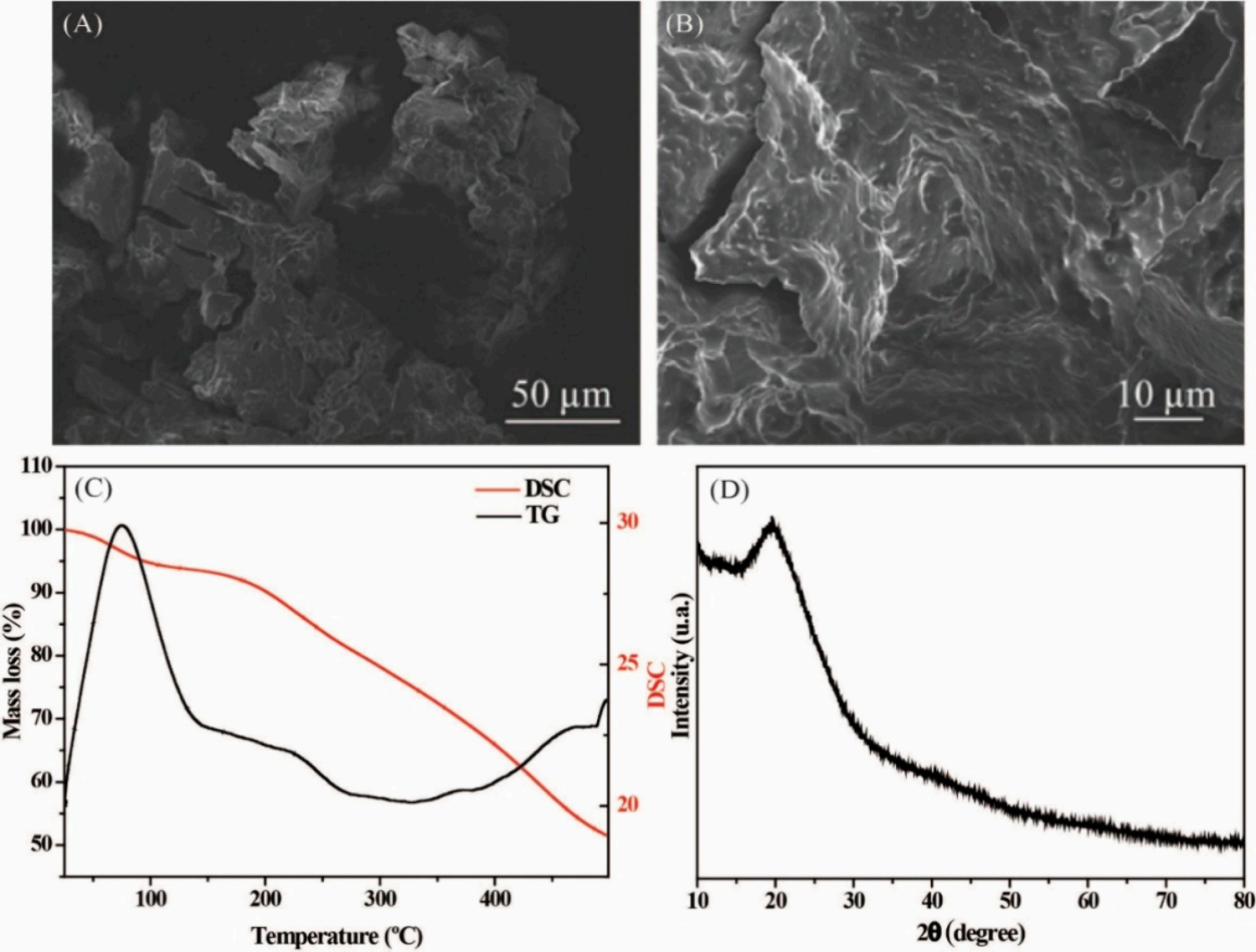
(A, B) SEM images of amorphous carbon particles produced from pumpkin seeds. (C) TGA and DSC of the carbon particles produced. (D) X-ray diffraction pattern of carbon particles.

Following, thermal stability was studied using TGA and Differential Scanning Calorimetry (DSC) techniques (Figure 1C). The TGA analysis revealed mass losses in three main stages: around ∼80 °C, ∼120–250 °C, and ∼250–450 °C. These events are attributed to water evaporation, decomposition of organic components from the pumpkin seed (possibly adsorbed or immobilized on the carbon surface), and residual carbonization, respectively.^25–28^ The material exhibits thermal stabilization beyond 450 °C. The DSC curve corroborated these transitions, presenting discrete endothermic and exothermic signals, characteristic of partially stabilized amorphous carbonaceous structures. Additionally, the XRD pattern exhibits a broad (Figure 1D), diffuse halo centered around 20°, corresponding to diffraction (002), with no sharp diffraction peaks.^29^ According to Bragg’s law, the corresponding interplanar distance is approximately 0.467 nm, greater than the interplanar distance of the graphite. This behavior is characteristic of disordered carbonaceous structures, typically found in nongraphitized or partially carbonized biomass-derived residues, often containing residual functional groups.^30^

To further investigate the textural and surface characteristics, the material was subjected to nitrogen adsorption studies. The isotherm shows extremely low N_2_ uptake, and the BET analysis yielded a surface area of 14.2 m^2^ g^–1^ with a total pore volume of only 0.00597 cm^3^ g^–1^, indicating very low porosity. The t-plot method revealed a micropore area of 5.76 m^2^ g^–1^ and an external surface area of 8.43 m^2^ g^–1^, confirming that most of the accessible area arises from the external surface of the particles rather than internal porosity. Moreover, the BET linear region exhibited poor correlation (r ≈ 0.18) and a low C constant (0.76), which is characteristic of materials with minimal nitrogen adsorption and confirms that the BET value should be interpreted only as an approximate indicator of low surface area. These features are consistent with nonporous or weakly porous amorphous carbon and agree with the compact morphology observed in the SEM images (Figure 1A,B). The full isotherm, BET plot, and all textural parameters are shown in the Supporting Information (Figures S1–S3 and Table S1). So far, through the characterization techniques performed, it has become evident that the material obtained is comprised of amorphous carbon particles, as expected; however, the chemical composition still needs to be investigated, mainly to evaluate endogenous self-doping, for which the FTIR (Figure 2) and XPS (Figure 3) techniques were used.

**Figure 2:**
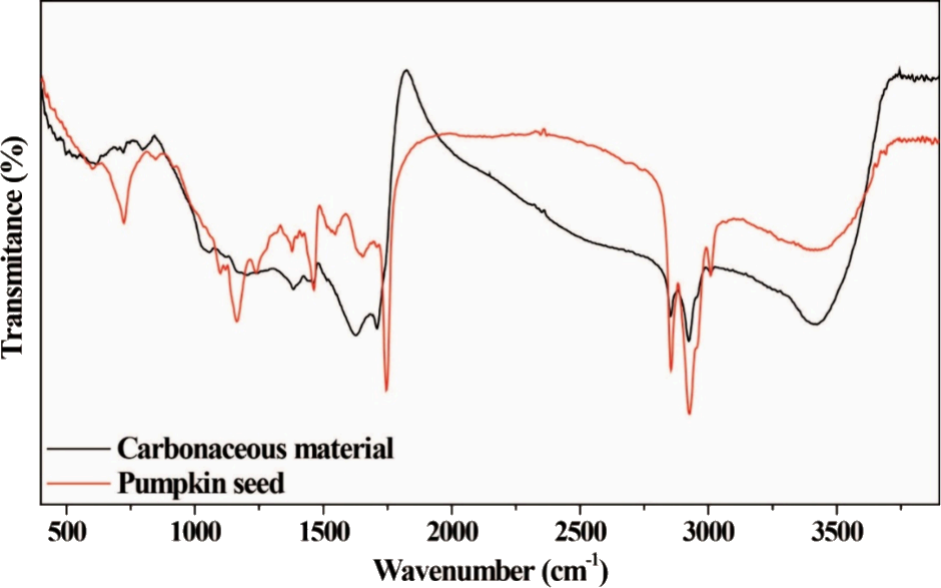
FTIR spectra of pumpkin seed and pumpkin seed-derived amorphous carbon.

**Figure 3:**
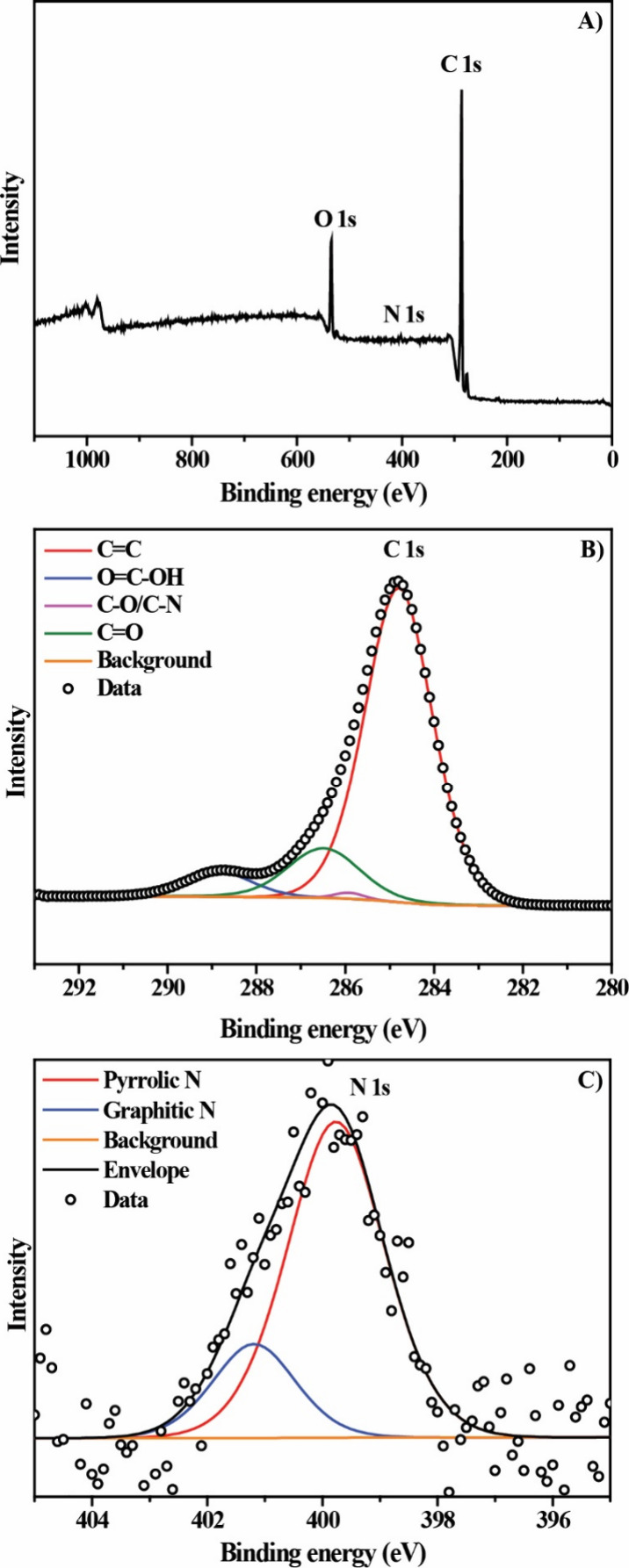
(A) XPS spectra survey and high-resolution of (B) C 1s and (C) N 1s of the pumpkin seed-derived amorphous carbon.

Figure 2 shows the FTIR spectra of the pumpkin seed and the pumpkin seed-derived amorphous carbon. As observed, the synthesized material exhibits several absorption bands, indicating the presence of various functional groups. Additionally, the synthesized material, when compared with the raw material, presents different and similar bands, suggesting that while some chemical characteristics of the precursor were retained, the synthesis process also introduced structural modifications, possibly resulting in new functional groups. Thus, to investigate and validate the self-doping process further, the XPS technique was used, as can be seen in Figure 3.

The survey spectrum (Figure 3A) shows the presence of the elements C, O, and N, with proportions of 90, 9, and 1%, respectively. The high resolution of C 1s (Figure 3B) shows the presence of four peaks centered at 248.8, 285.9, 286.5, and 288.8 eV that can be assigned to C=C (79.4%),^31,32^ C–O/C–N (0.7%),^32,33^ C=O (13.8%),^33^ and O=C–OH (6.1%),^33^ respectively. These results indicate that the material is predominantly composed of graphitic-like carbon domains, while a significant fraction of oxygen-containing functional groups remains on the surface, which may act as active sites for oxygen adsorption and stabilization of reaction intermediates during the ORR. Additionally, the N 1s (Figure 3C) reveals the presence of pyrrolic and graphite nitrogen,^34–37^ even with low nitrogen concentrations. This result demonstrates that nitrogen was successfully introduced into the carbon structure, demonstrating that the endogenous self-doping process was successful, since nothing other than pumpkin seeds and phosphoric acid was added to the reaction medium. This phenomenon is primarily due to the rich chemical composition of pumpkin seeds, which contain carbohydrates and proteins rich in C, O, and nitrogen compounds.^17–20^ These results show that during the synthesis process, dense and amorphous carbon particles were obtained with endogenous self-doping.

For initial studies of the catalytic activity of the material, electrochemical measurements were performed using an RDE in a potential window of 1.0 to 0.22 V vs RHE with a rotational speed of 1600 rpm. As shown in Figure 4A, for the carbonaceous material, an excellent performance is observed for the ORR reaction, with an onset potential (E_onset_) of 0.738 V and a half-wave potential (*E*_1/2_) of 0.403 V. For Vulcan, the E_onset_ and *E*_1/2_ were 0.72 and 0.47 V, respectively, relatively higher values than the electrocatalyst under study. As expected, carbonaceous material has a lower current density (j) than Vulcan, whose values are −1.5 mA·cm^–2^ and −3.28 mA·cm^–2^, respectively. To better understand the reaction to the electrocatalyst, ORR polarization curves at various rotational speeds, ranging from 400 to 2500 rpm, were performed in RDE, as shown in Figure S4A,B. For both materials, with the increase in the rotation rate of the RDE, there is an improvement in mass transport and, therefore, in its current density.

**Figure 4:**
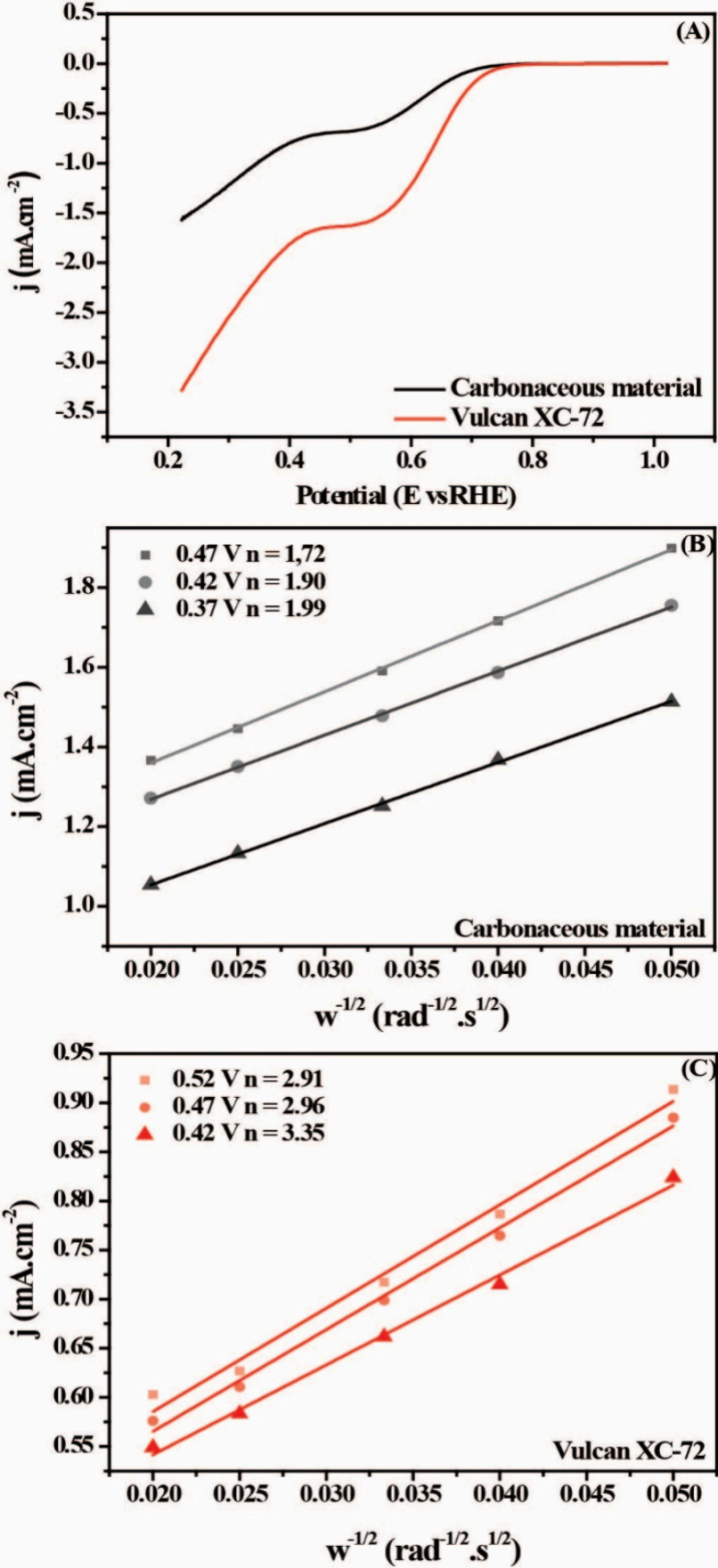
(A) LSV curves of carbonaceous material and Vulcan-XC 72 in 0.1 M O_2_-saturated KOH. Corresponding K-L plots at different potentials derived from (B) RRDE measurements for carbonaceous material and (C) Vulcan-XC 72.

The K-L graphs were obtained by treating the polarization curves at different rotation rates (Figure 4B,C). K-L to E slopes smaller than 0.52 V have good linearity. The carbonaceous material graph is parallel to the theoretical value of 2e^–^, predicting excellent ORR activity for the desired mechanism, while the Vulcan slopes are more parallel to that of 3e^–^ (Figure S5). When the number of electrons transferred (n) was calculated, the pumpkin seed-derived amorphous carbon presented values of 1.99 and 3.02 for the Vulcan.

To better evaluate the ORR pathways for carbonaceous material, measurements using RRDE were performed at KOH 0.1 M at 1600 rpm. As shown in Figure 5A, carbonaceous material presented a value of disc current density of 1.57 mA·cm^–2^ for O_2_ reduction and a ring current density of 0.24 mA·cm^–2^ for H_2_O_2_ oxidation, while for Vulcan these values were 3.28 mA·cm^–2^ and 0.24 mA·cm^–2^ for disc and ring current, respectively. N and H_2_O_2_% as a function of potential are present in Figure 5B,C. The average electron transfer number is 2.16 with the potential decreasing from 1.0 to 0.22 V, indicating that the two-electron pathway is in this potential range. The average H_2_O_2_% is approximately 91.81% in the range of 1.0 to 0.22 V. For Vulcan, the n increased significantly as the potential advanced, but the average value remained at 2.48, showing that in some potentials, the material is less favorable for the 2e^–^ mechanism. H_2_O_2_% corroborates with the data from n, showing that the selectivity ranged from 93.77 to 46.51%, showing a significant drop in the production potential of Vulcan. Figure 5D demonstrates that the 2e^–^ ORR Faradaic efficiency on the carbonaceous material electrode reaches a maximum of 115% and remains consistent at a value of approximately 62.2% in a potential window of 1.0 to 0.22 V. In contrast, the Vulcan EF is substantially lower, with a maximum value reaching 119% and falling sharply to 30.3%.

**Figure 5:**
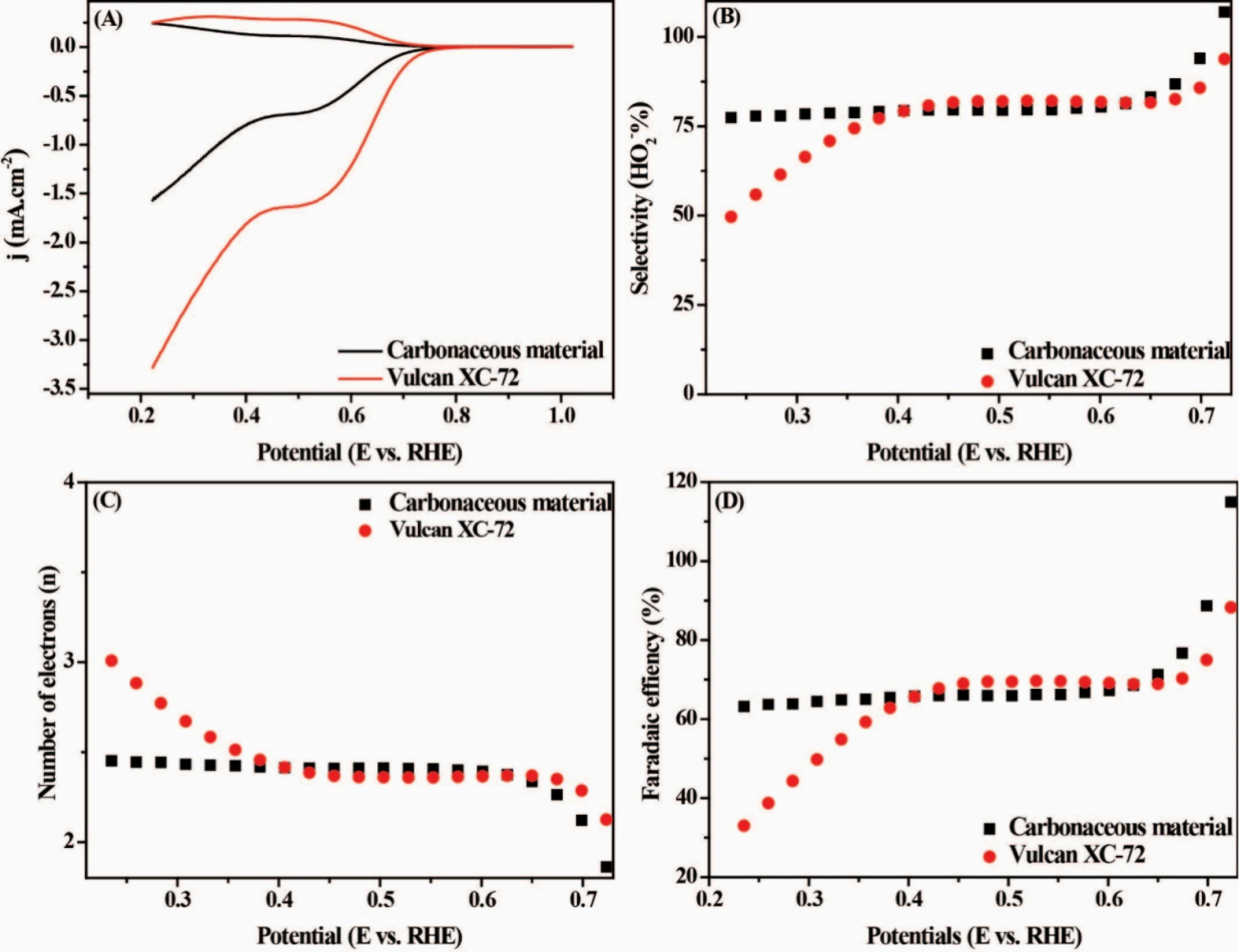
(A) LSVs of RRDE at 1600 rpm in O_2_-saturated 0.1 M KOH at 5 mV.s^–1^. (B) H_2_O_2_ selectivity, (C) n, and (D) EF as a function of the applied potential.

Such results are possibly due to the presence of oxygenated functional groups on the surface of carbonaceous material, which act as specific active sites that favor the 2e^–^ mechanism, as they act as moderately strong adsorption sites of reactive species, such as the *OOH radical, stabilizing them in a way that favors the premature release of HO^2–^ before the O–O bond break, necessary for the formation of OH^–^.^37,38^ In addition, the amorphous nature of carbon, with its defects, high density of edges and disordered regions, generates less intense surface adsorption and delays the subsequent reduction steps, creating conditions conducive to the 2e^^–^^ pathway.^39^ In addition, nitrogen doping in pyrrolic and graphitic forms modulates the local electron density to reinforce this selectivity: N-pyrrolic, present at faulty edges and sites, increases affinity for the *OOH radical without promoting O–O bond cleavage, while N-graphitic, incorporated into the basal lattice, improves the electrical conductivity of the material without significantly altering the selectivity for the 2e^^–^^ pathway. Considering that the nitrogen content is relatively low, the oxygen-containing surface functionalities likely play the dominant role in determining the catalytic behavior, acting as the primary active sites for the 2e^–^ ORR pathway. In this context, the nitrogen species are better interpreted as providing a synergistic electronic modulation of these oxygen-containing sites, tuning the local charge distribution and adsorption strength of reaction intermediates, thereby reinforcing the selectivity toward H_2_O_2_ formation rather than acting as the main active centers.

The Tafel plots (Figure 6A) were calculated from the experimental data of the RRDE. It is shown that the slopes were 60.58 mV·dec^–1^ and 63.96 mV·dec^–1^ for carbonaceous material and Vulcan, respectively. The results indicate a rapid oxygen reduction kinetics under alkaline conditions. Considering that the stability of the catalyst is an important factor for practical applications, the chronoamperometry technique was performed at 0.72 V at 1600 rpm for 7200 s. The H_2_O_2_% and n are shown in Figure 6B,C. The n for carbonaceous material during the reaction remained less than 2.40, implying the 2e^–^ ORR pathway for the electrocatalyst. In addition, selectivity remained significantly high after 7200 s, stabilized at 82.57%. We then quantified the H_2_O_2_ accumulated in the electrolyte during the ORR process using an ultraviolet–visible spectrophotometer (UV–vis) to investigate the H_2_O_2_ yield rate (Figure 6D). For our electrocatalyst, the yield rate remained unchanged throughout the potentials investigated, with a maximum H_2_O_2_ yield at 0.72 V vs RHE, with 612.7 mmol.g^–1^.h^–1^, showing an evident advantage in comparison, in the hourly yield, with evident studies in the literature, as shown in Table 1.

**Figure 6:**
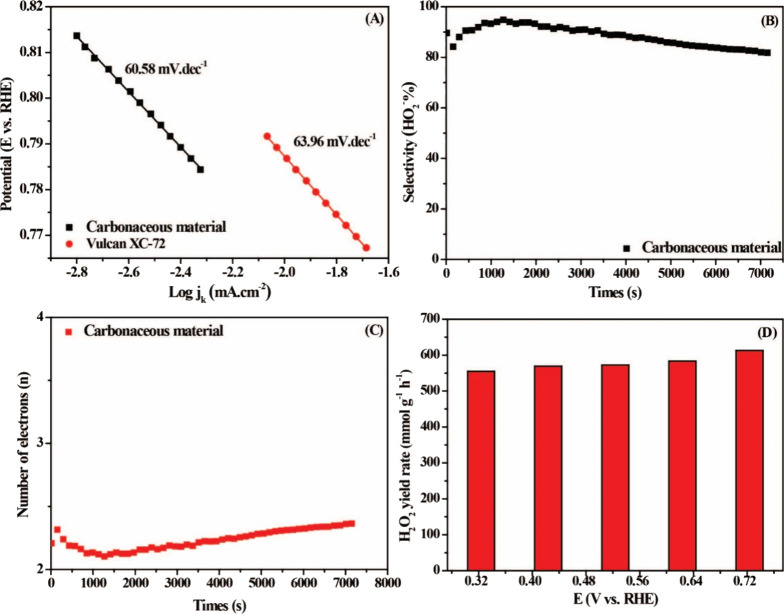
(A) Tafel plots for carbonaceous material (black) and Vulcan XC-72 (red). (B) H_2_O_2_ selectivity of carbonaceous material, determined from chronoamperometry measurements. (C) Electron transfer number (n) of carbonaceous material. (D) H_2_O_2_ production rate of carbonaceous material, normalized by catalyst loading and electrode geometric area.

**Table 1:** Comparison 2e^–^ ORR Performance under Alkaline Conditions Reported Work

catalysts	H_2_O_2_ yield rate (mmol g^–1^ h^–1^)	**reference**
Pd_*x*_-NC	∼14	^39^
O–CNT	120	^40^
g-N-CNH	54	^41^
rGO/PEI	110	^42^
pumpkin seed-derived amorphous carbon	612.7	this work

The electrochemical results indicate that the amorphous carbon derived from pumpkin seeds does not surpass Vulcan XC-72 in terms of geometric current density. However, it exhibits comparable H_2_O_2_ selectivity in the low-overpotential region and maintains stable 2e^–^ ORR behavior across the investigated potential range. Importantly, rather than aiming to outperform commercial carbon materials, this study demonstrates that a sustainably derived biomass-based material can reach electrochemical performance comparable to widely used benchmark carbons, highlighting the potential of biomass valorization strategies for the development of cost-effective electrocatalysts for hydrogen peroxide production.

EIS was conducted to investigate the electrochemical properties of the electrocatalyst (Figure 7). The double-layer electrochemical capacitance (Cdl), commonly proportional to the ECSA, was determined from the EIS data recorded in the nonfaradaic region by eqs 6 and 7. In the nonfaradaic region, the Cdl value for the pumpkin seed-derived amorphous carbon material was 0.48 μF·cm–2, while the calculated ECSA was 0.0108 cm2. These values indicate a highly active surface from an electrochemical point of view, which may be directly related to the improved performance of the material against ORR. Additionally, the EIS showed a significant reduction in the load transfer resistance (Rct), from 167 kΩ at 0 Vref to only 430 Ω at 0.65 V vs RHE. This significant decrease suggests an improvement in electron conduction properties and electron transfer kinetics. The combination of a relatively high double-layer capacitance with a significantly reduced charge resistance reinforces the hypothesis that the pumpkin seed-derived amorphous carbon material has an active surface that favors adsorption and oxygen reduction, evidencing the material’s potential as an efficient electrocatalyst, with good accessibility of active sites and favorable kinetics for the reaction.

**Figure 7:**
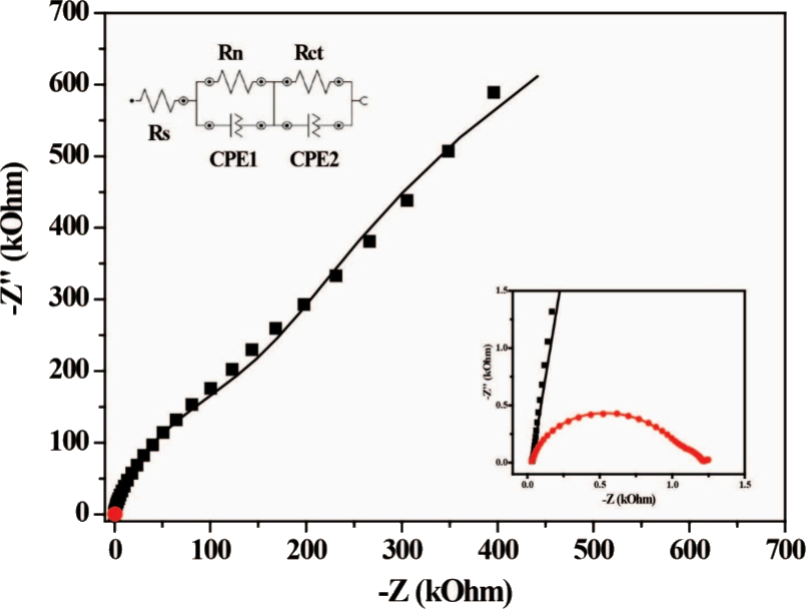
EI spectra of ORR recorded with pumpkin seed-derived amorphous carbon in oxygen-saturated 0.1 M KOH solution at 1600 rpm. The inset shows the complete Nyquist plot.

## 4 Conclusions

In this study, amorphous carbon particles were successfully synthesized from pumpkin seed biomass as a byproduct of carbon quantum dot production, establishing a sustainable and circular approach to biomass utilization. Structural and surface characterizations confirmed the formation of dense, nongraphitized carbon containing endogenous nitrogen doping derived from the intrinsic composition of the precursor. Electrochemical analyses demonstrated that the material exhibits high selectivity toward the two-electron oxygen reduction pathway, with an average H_2_O_2_ selectivity of 91.8% and an electron transfer number close to 2. This behavior is associated with the presence of oxygen- and nitrogen-containing functional groups, which act as active sites capable of stabilizing the *OOH intermediate and promoting the 2e^–^ pathway. In this context, nitrogen species are better interpreted as providing a synergistic electronic modulation of these oxygen-based active sites, tuning the local electronic structure and adsorption strength of reaction intermediates.

The catalyst also exhibited long-term stability and a high H_2_O_2_ production rate of 612.7 mmol g^–1^ h^–1^. Although the material does not surpass Vulcan XC-72 in terms of geometric current density, it demonstrates comparable selectivity, establishing it as a technically viable and sustainable alternative for H_2_O_2_ electrosynthesis. In summary, these results demonstrate that pumpkin seed–derived amorphous carbon is a promising, low-cost, and metal-free electrocatalyst for selective H_2_O_2_ production via ORR, highlighting its potential for sustainable energy and environmental applications through effective biomass waste valorization.

## Supplementary Material



## Data Availability

Data used is available throughout the manuscript text.

## Notes

BET analysis, textural parameters, adsorption isotherms, T-plot analysis, and ORR polarization curves for pumpkin seed-derived carbon and Vulcan XC-72 (PDF)

## References

[1] Hayat A., Ajmal Z., Alzahrani A. Y. A., Moussa S. B., Khered M., Almuqati N., Alshammari A., Al-Hadeethi Y., Ali H., Orooji Y. (2025). The Photocatalytic H2O2 Production: Design Strategies,
Photocatalyst Advancements, Environmental Applications and Future
Prospects.. Coord. Chem. Rev..

[2] Jiang Z., Li C., Qi F., Wang Z., Liu Y., Li F., Wang H., Bian Z., Zhu M., Kumirska J., Siedlecka E. M. (2025). A Review on Photocatalytic Hydrogen
Peroxide Production
from Oxygen: Material Design, Mechanisms, and Applications.. ACS Appl. Mater. Interfaces.

[3] He Y., Zhang H. J., Yang Y., Xue Y. (2024). Progress of Metal-Free
Carbon Alloy Electrocatalysts for Hydrogen Peroxide by Two-Electron
Oxygen Reduction Reaction.. J. Electroanal.
Chem..

[4] Sun Y., Fan K., Li J., Wang L., Yang Y., Li Z., Shao M., Duan X. (2024). Boosting Electrochemical Oxygen Reduction
to Hydrogen Peroxide Coupled with Organic Oxidation. Nat. Commun..

[5] Wang Y., Wang Y., Zhao J., Tang T., Lv B., Chang Y., Hu T., Zhang J., Luo E., Jia J. (2024). Optimizing Heteroatom Doping for Efficient Hydrogen Peroxide Production
via Oxygen Reduction.. ACS Sustainable Chem.
Eng..

[6] Oliveira E. S., Pereira F. S., Martins J. S., Silva F. A. e, Alcântara A., Liu L., de Almeida J. M. A. R., Romano P. N., Tanaka A. A., Rodrigues T. S., Garcia M. A. S. (2025). Highly Selective Hydrogen Peroxide
Production Using an AgPd-Based Electrocatalyst with Ultralow Pd Loading.. ACS Omega.

[7] Jalalah M., Han H. S., Nayak A. K., Harraz F. A. (2023). Biomass-Derived
Metal-Free Porous Carbon Electrocatalyst for Efficient Oxygen Reduction
Reactions.. J. Taiwan Inst Chem. Eng..

[8] Wang G., Peng H., Qiao X., Du L., Li X., Shu T., Liao S. (2016). Biomass-Derived Porous
Heteroatom-Doped Carbon Spheres
as a High-Performance Catalyst for the Oxygen Reduction Reaction.. Int. J. Hydrogen Energy.

[9] Wang Y., Zhong H., Yang W., Feng Y., Alonso-Vante N. (2023). Recent Advances
with Biomass-Derived Carbon-Based Catalysts for the High-Efficiency
Electrochemical Reduction of Oxygen to Hydrogen Peroxide.. Advanced Energy and Sustainability Research.

[10] Cao Y., Sun Y., Zheng R., Wang Q., Li X., Wei H., Wang L., Li Z., Wang F., Han N. (2023). Biomass-Derived
Carbon Material as Efficient Electrocatalysts for the Oxygen Reduction
Reaction. Biomass Bioenergy.

[11] Abu N., Chinnathambi S., Kumar M., Etezadi F., Bakhori N. M., Zubir Z. A., Md Salleh S. N., Shueb R. H., Karthikeyan S., Thangavel V., Abdullah J., Pandian G. N. (2023). Development of Biomass
Waste-Based Carbon Quantum Dots and Their Potential Application as
Non-Toxic Bioimaging Agents. RSC Adv..

[12] Kumar K., Kumar R., Kaushal S., Thakur N., Umar A., Akbar S., Ibrahim A. A., Baskoutas S. (2023). Biomass Waste-Derived
Carbon Materials for Sustainable Remediation of Polluted Environment:
A Comprehensive Review. Chemosphere.

[13] de
Sousa Oliveira E., Figueredo A. L., Gothe M. L., Vidinha P., Tanaka A. A., Garcia M. A. S. (2025). Optimizing Metal-Free Phenanthroline-Assisted
Nitrogen-Doped Reduced Graphene Oxide for Enhanced Oxygen Reduction
Reaction: An Experimental Design and Performance Study. Electrocatalysis.

[14] Das S., Ghosh S., Kuila T., Murmu N. C., Kundu A. (2022). Biomass-Derived
Advanced Carbon-Based Electrocatalysts for Oxygen Reduction Reaction. Biomass.

[15] Sun L., Sun L., Huo L., Zhao H. (2023). Promotion of the Efficient Electrocatalytic
Production of H2O2 by N,O- Co-Doped Porous Carbon. Nanomaterials.

[16] Ningthoujam M. D., Gaibimei P., Devi M., Prasad R. V., Palmei G. (2018). Physico-Chemical
Characterisation of Pumpkin Seeds. Int. J. Chem.
Stud.

[17] Saxena D., Sharma U., Gupta S., Mahajan S. (2022). Pumpkin Seeds as a
Power House of Nutrition: A Review. Indian J.
Nutr Diet.

[18] Rezig L., Chouaibi M., Msaada K., Hamdi S. (2012). Chemical Composition
and Profile Characterisation of Pumpkin (Cucurbita Maxima) Seed Oil. Ind. Crops Prod.

[19] Seymen M., Uslu N., Türkmen Ö., Al Juhaimi F., Özcan M. M. (2016). Chemical Compositions and Mineral
Contents of Some
Hull-Less Pumpkin Seed and Oils. JAOCS.

[20] Magno
Paiva V., Massafra de Oliveira S., Muniz da
Silva de Almeida C., Rodrigues de Araujo J., Soares Archanjo B., D'Elia E. (2024). Pumpkin (Cucurbita Maxima) Seed-Derived Nitrogen, Phosphorus,
and Sulfur Carbon Quantum Dot as an Inhibitor of Corrosion for Mild
Steel in HCl Solution. J. Mater. Res. Technol..

[21] Zhao Q., An J., Wang S., Qiao Y., Liao C., Wang C., Wang X., Li N. (2019). Superhydrophobic Air-Breathing Cathode
for Efficient Hydrogen Peroxide Generation through Two-Electron Pathway
Oxygen Reduction Reaction. ACS Appl. Mater.
Interfaces.

[22] Xia Y., Zhao X., Xia C., Wu Z. Y., Zhu P., Kim J. Y., Bai X., Gao G., Hu Y., Zhong J., Liu Y., Wang H. (2021). Highly Active
and Selective
Oxygen Reduction to H2O2 on Boron-Doped Carbon for High Production
Rates. Nat. Commun..

[23] Salgado M. D. F., Abioye A. M., Junoh M. M., Santos J. A. P., Ani F. N. (2018). Preparation
of Activated Carbon from Babassu Endocarpunder Microwave Radiation
by Physical Activation. IOP Conf Ser. Earth
Environ. Sci..

[24] Das O., Kim N. K., Hedenqvist M. S., Lin R. J. T., Sarmah A. K., Bhattacharyya D. (2018). An Attempt
to Find a Suitable Biomass for Biochar-Based
Polypropylene Biocomposites. Environ. Manage.

[25] Yi Q., Qi F., Cheng G., Zhang Y., Xiao B., Hu Z., Liu S., Cai H., Xu S. (2013). Thermogravimetric Analysis of Co-Combustion
of Biomass and Biochar. J. Therm Anal Calorim.

[26] Meysami M., Rabie A., Najafabadi R. A., Meysami A., Isfahani T. (2025). Comparative
Thermal and Structural Analysis of Biochar from Rapeseed Meal and
Fraxinus Excelsior Sawdust. Results in Engineering.

[27] Nda-Umar U. I., Ramli I., Muhamad E. N., Taufiq-Yap Y. H., Azri N. (2022). Synthesis and Characterization of
Sulfonated Carbon Catalysts Derived
from Biomass Waste and Its Evaluation in Glycerol Acetylation. Biomass Convers. Biorefin..

[28] Wu H., Dong Z., Sun J., Ding K. (2024). Boosting the Adsorption
Capacity of Activated Carbon Prepared from Amygdalus Communis Shells
Using Physicochemical Co-Activation Method. Biomass Convers Biorefin.

[29] Wang H., Liu J., Ju W., Xu X., Chen J. (2024). Nitrogen-Doped Hollow
Carbon Sphere Composite Mn3O4 as an Advanced Host for Lithium-Sulfur
Battery. Sci. Rep.

[30] Zhang C., Ren X., Kou L., Zhang X., Wang R., Xie L., Fan C. (2021). Facile Synthesis
of Nitrogen-Rich Porous Carbon Spheres Assisted
by NaNH2 as a Bifunctional Activator and Nitrogen Source for CO2 Capture. J. Environ. Chem. Eng..

[31] Nguyen K. G., Baragau I. A., Gromicova R., Nicolaev A., Thomson S. A. J., Rennie A., Power N. P., Sajjad M. T., Kellici S. (2022). Investigating
the Effect of N-Doping on Carbon Quantum Dots Structure, Optical Properties
and Metal Ion Screening. Sci. Rep.

[32] Zhang L., Li B., Zhou Y., Wu Y., Le T., Sun Q. (2023). Green Synthesis
of Cow Milk-Derived Carbon Quantum Dots and Application for Fe3+ Detection. J. Solgel Sci. Technol..

[33] Ye D., Leung K. C., Niu W., Duan M., Li J., Ho P. L., Szalay D., Wu T. S., Soo Y. L., Wu S., Tsang S. C. E. (2024). Active Nitrogen Sites on Nitrogen Doped Carbon for
Highly Efficient Associative Ammonia Decomposition. iScience.

[34] Yokwana K., Ntsendwana B., Nxumalo E. N., Mhlanga S. D. (2023). Recent
Advances
in Nitrogen-Doped Graphene Oxide Nanomaterials: Synthesis and Applications
in Energy Storage, Sensor Electrochemical Applications and Water Treatment. J. Mater. Res..

[35] Boas C. R. S. V., Focassio B., Marinho E., Larrude D. G., Salvadori M. C., Leão C. R., dos Santos D. J. (2019). Characterization of Nitrogen Doped
Graphene Bilayers Synthesized by Fast, Low Temperature Microwave Plasma-Enhanced
Chemical Vapour Deposition. Sci. Rep..

[36] Yu A., Liu S., Yang Y. (2024). Recent Advances in Electrosynthesis of H 2 O 2 via
Two-Electron Oxygen Reduction Reaction. Chem.
Commun..

[37] Albashir A. I. M., Lu X., Dai X., Qi W. (2024). Effects of Porous Structure
and Oxygen Functionalities on Electrochemical Synthesis of Hydrogen
Peroxide on Ordered Mesoporous Carbon. Communications
Chemistry.

[38] An J., Feng Y., Zhao Q., Wang X., Liu J., Li N. (2022). Electrosynthesis of H2O2 through a Two-Electron Oxygen Reduction
Reaction by Carbon Based Catalysts: From Mechanism, Catalyst Design
to Electrode Fabrication. Environmental Science
and Ecotechnology.

[39] Wang N., Zhao X., Zhang R., Yu S., Levell Z. H., Wang C., Ma S., Zou P., Han L., Qin J., Ma L., Liu Y., Xin H. L. (2022). Highly
Selective
Oxygen Reduction to Hydrogen Peroxide on a Carbon-Supported Single-Atom
Pd Electrocatalyst. ACS Catal..

[40] Lu Z., Chen G., Siahrostami S., Chen Z., Liu K., Xie J., Liao L., Wu T., Lin D., Liu Y., Jaramillo T. F., Nørskov J. K., Cui Y. (2018). High-Efficiency Oxygen
Reduction to Hydrogen Peroxide Catalysed by Oxidized Carbon Materials. Nat. Catal.

[41] Iglesias D., Giuliani A., Melchionna M., Marchesan S., Criado A., Nasi L., Bevilacqua M., Tavagnacco C., Vizza F., Prato M., Fornasiero P. (2018). N-Doped Graphitized
Carbon Nanohorns as a Forefront Electrocatalyst in Highly Selective
O2 Reduction to H2O2. Chem..

[42] Xiao X., Wang T., Bai J., Li F., Ma T., Chen Y. (2018). Enhancing the Selectivity of H 2
O 2 Electrogeneration by Steric
Hindrance Effect. ACS Appl. Mater. Interfaces.

