# Supplementary material for: Biomass-Derived Amorphous Carbon with Intrinsic Nitrogen Doping for Hydrogen Peroxide Electrosynthesis
Source: ACS Omega. 2026 Apr 27;11(18):26726–35. doi: 10.1021/acsomega.5c13403 (PMC13177241; doi:10.1021/acsomega.5c13403)
Supplement: Supplementary file 1 [file ao5c13403_si_001.pdf]

Supporting Information

Biomass-Derived Amorphous Carbon with Intrinsic Nitrogen Doping for High-Performance Hydrogen Peroxide Electrosynthesis

Fellipe dos Santos Pereira[1], *Victor Magno Paiva*[1], *Agnes Candido Teixeira*[2], *Marcelo Eduardo Huguenin Maia da Costa*[3], *Emanuel do Couto-Pessanha*[4], *Bojan A. Marinkovic*[4], *Natasha Midori Suguihiro*[5], *Pedro Nothaft Romano,*[1,2,6] *Marco Aurélio Suller Garcia,*[2,*] *João Monnerat Araújo Ribeiro de Almeida,*[1] *and Eliane D'Elia*[1,*]

[1]*Department of Inorganic Chemistry, Universidade Federal do Rio de Janeiro (UFRJ), Avenida Athos da Silveira Ramos, 149, Cidade Universitária, 21941-909 Rio de Janeiro, Brazil.*

[2]*Nanotechnology Engineering Program, UFRJ, Avenida Horácio Macedo, 2030, Cidade Universitária, 21941-972 Rio de Janeiro, Brazil.*

[3]*Department of Physics Pontifícia Universidade Católica do Rio de Janeiro (PUC-RJ), Rua Marquês de São Vicente, 225, Gávea, 22451-900 Rio de Janeiro, Brazil.*

[4]*Department of Chemical and Materials Engineering, Pontifical Catholic University of Rio de Janeiro (PUC-Rio), 22453-900, Rio de Janeiro, RJ, Brazil*

[5]*Department of Nanotecnology, UFRJ - Duque de Caxias, Professor Geraldo Cidade, Rodovia Washington Luiz, 19593, 25240-005 Duque de Caxias, Brazil.*

[6]*Programa de Pós-Graduação em Química (PGQu),UFRJ, Avenida Athos da Silveira Ramos, 149, Rio de Janeiro 21941-909 Rio de Janeiro, Brazil.*

**marcosuller@pent.coppe.ufrj.br*
*eliane@iq.ufrj.br

Table S1. BET Surface Area and Textural Parameters of Sample.

Parameter	Value	Method
**Surface area (BET)**	**14.19 m²/g**	Multipoint BET
**Total pore volume**	**0.00597 cm³/g**	P/Po = 0.989
**Average pore radius**	**0.84 nm**	BET method
**Micropore volume**	**–0.00265 cm³/g**	t-plot (indicates negligible microporosity)
**Micropore area**	**5.76 m²/g**	t-plot
**External surface area**	**8.43 m²/g**	t-plot
**DFT pore volume**	**0.0055 cm³/g**	NLDFT
**BJH pore volume**	**0.00750 cm³/g**	BJH desorption

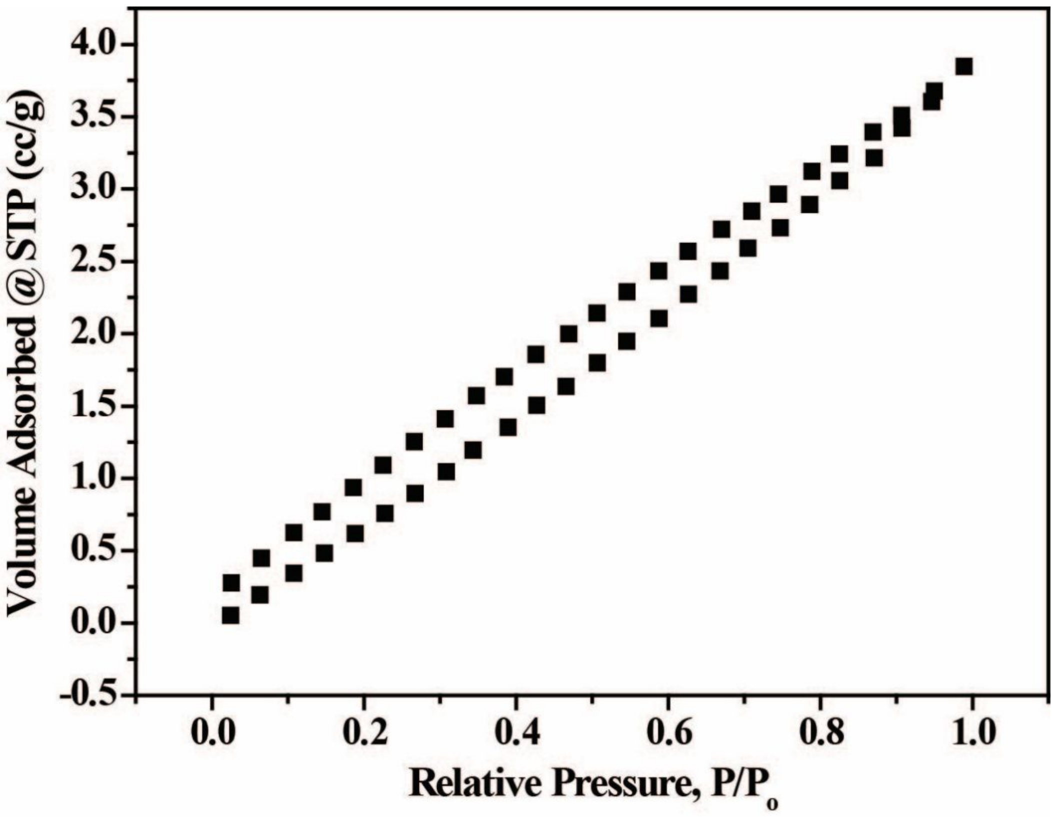

Figure S1. BET Surface Area and Textural Parameters of pumpkin seed-derived amorphous carbon.

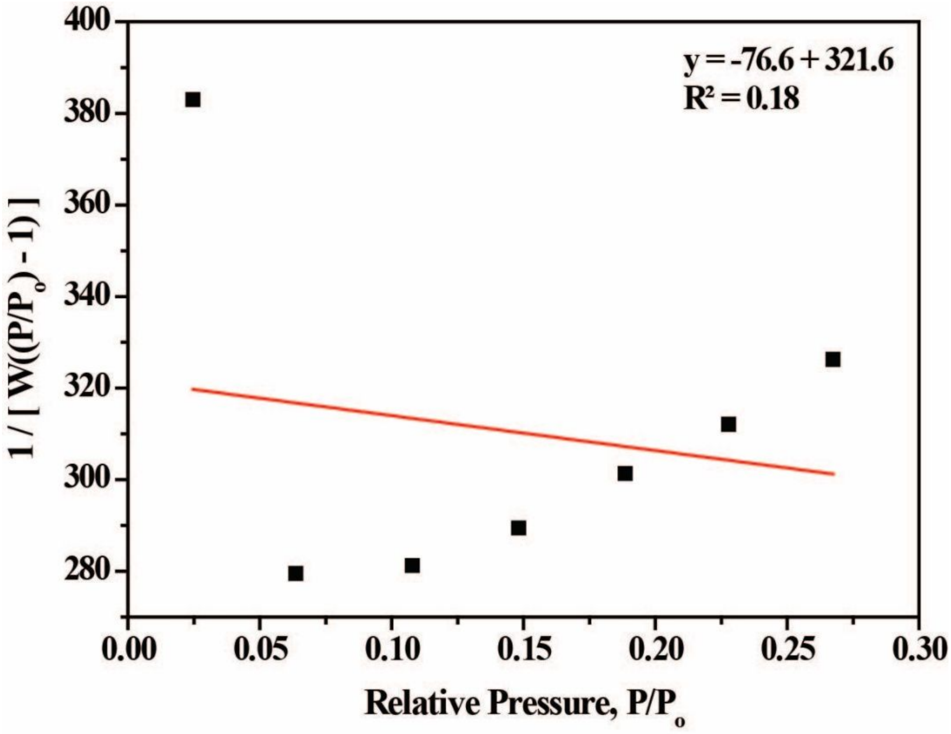

Figure S2. BET linear plot used for the determination of the surface area of the pumpkin seed-derived amorphous carbon.

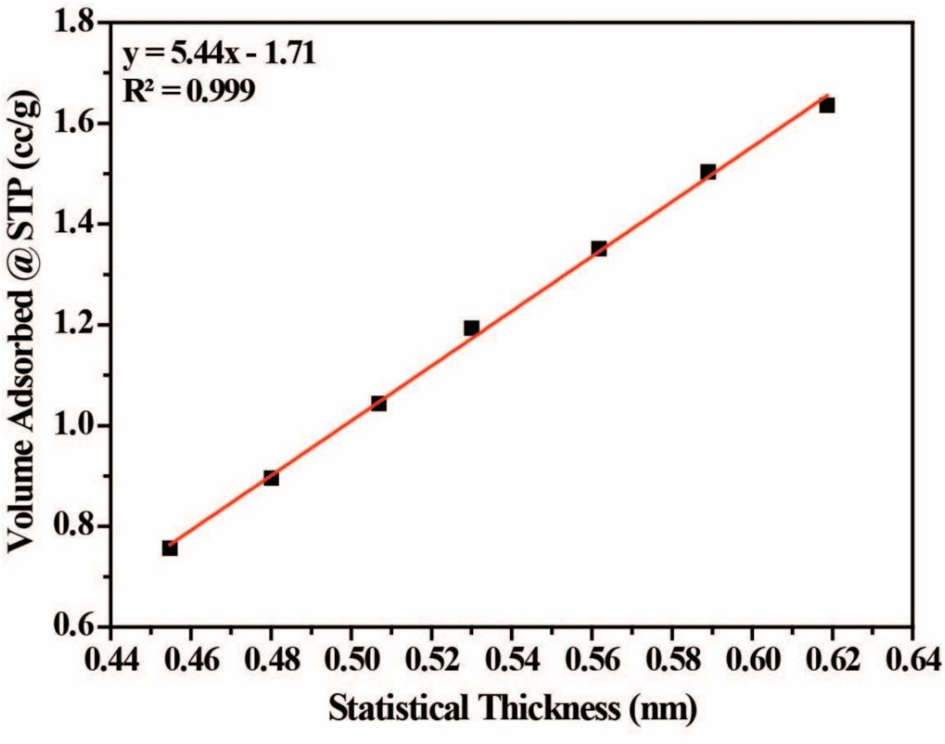

Figure S3. T-plot of pumpkin seed-derived amorphous carbon used to determine micropore area and external surface area.

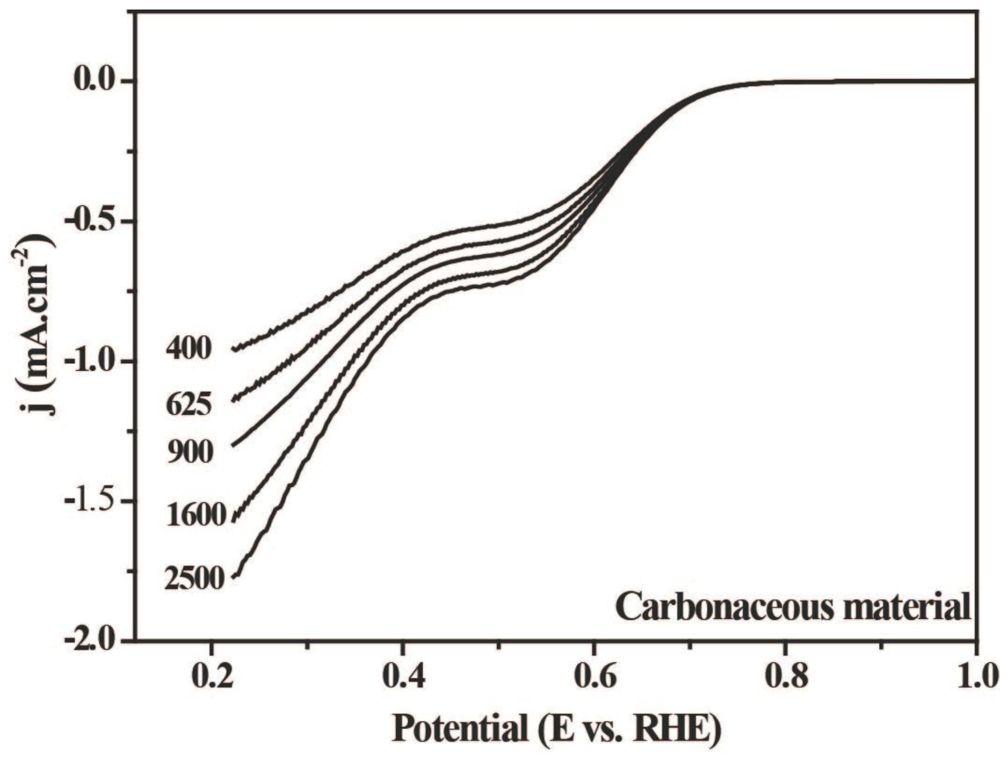

Figure S4. ORR polarization curves of pumpkin seed-derived amorphous carbon at the various rotation speeds at sweep rate 10 mV s^{-1} in O_2-saturated 0.1 M KOH solution.

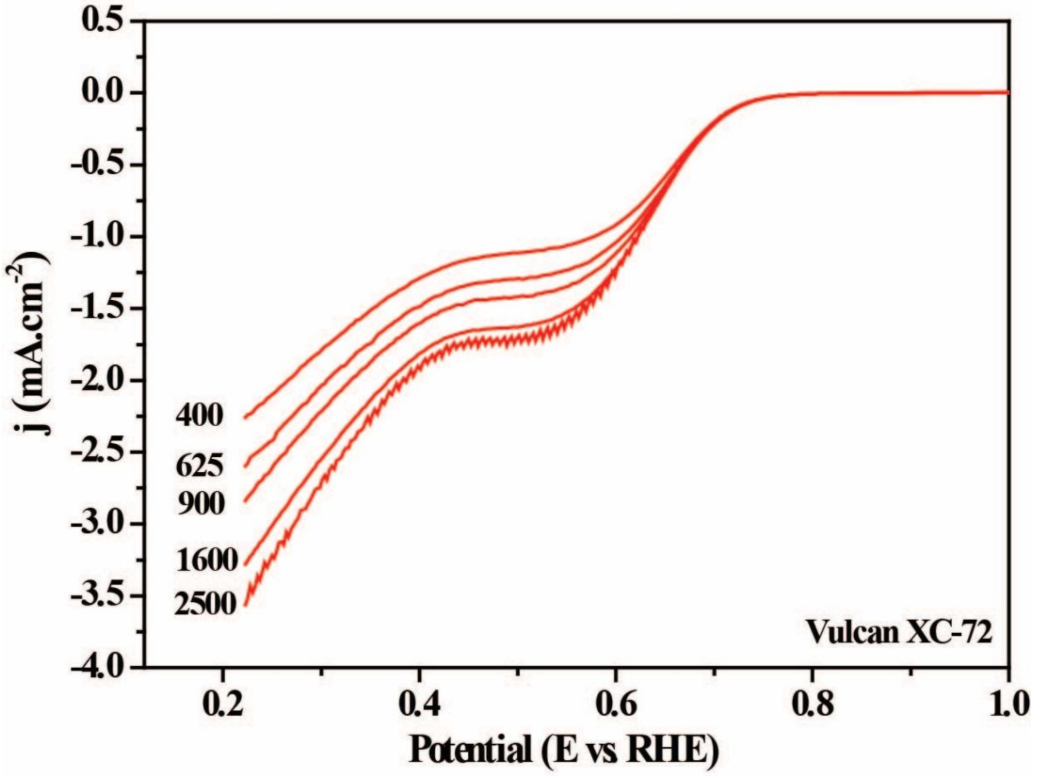

Figure S5. ORR polarization curves of Vulcan XC-72 at the various rotation speeds at sweep rate 10 mV s −1 in O_2-saturated 0.1 M KOH solution.
